# Literature review of complementary and alternative therapies: using text mining and analysis of trends in nursing research

**DOI:** 10.1186/s12912-024-02172-9

**Published:** 2024-08-01

**Authors:** Jihye Nam, Hyejin Lee, Seunghyeon Lee, Hyojung Park

**Affiliations:** College of Nursing, Ewha Womans University, 52, Ewhayeodae-Gil, Seodaemun-Gu, Seoul, 03760 South Korea

**Keywords:** Alternative, Complementary, Nursing, Text network, Trend

## Abstract

**Purpose:**

This study aimed to review the literature on complementary and alternative therapies, utilizing text mining and trend analysis in nursing research. As CAM becomes increasingly prevalent in healthcare settings, a comprehensive understanding of the current research landscape is essential to guide evidence-based practice, inform clinical decision-making, and ultimately enhance patient outcomes.

**Method:**

This study aimed to identify CAM-related literature published from 2018 to 2023. Using the search terms 'complementary therap*', 'complementary medicine', 'alternative therap*', and 'alternative medicine', we performed a comprehensive search in eight databases, including EMBASE, Cochrane Central, PubMed Central, Korea Education and Research Information Service (RISS), Web of Science, KMbase, KISS, and CINAHL. From the text network and topic modeling analysis of 66,490 documents, 15 topics were identified. These topics were classified into two nursing-related topics through an academic classification process involving three doctors with doctoral degrees, three nurses, and three pharmacists. Based on the classified topics, research trends were comparatively analyzed by re-searching the database for 12 nursing and 22 non-nursing literature.

**Result:**

This study found that in nursing literature, yoga is used to improve mental symptoms such as stress and anxiety. In non-nursing literature, most of the experimental studies on complementary and alternative therapies were conducted in a randomized manner, confirming that a variety of physiological and objective indicators were used. Additionally, it was discovered that there were differences in the diversity of research subjects and research design methods for the same intervention method. Therefore, future research should focus on broadening the scope of subjects and measurement tools in nursing studies. Additionally, such studies should be conducted with randomization and generalizability in the experimental design in mind.

**Conclusion:**

This study employed text network analysis and text mining to identify domestic and international CAM research trends. Our novel approach combined big data-derived keywords with a systematic classification method, proposing a new methodological strategy for trend analysis. Future nursing research should focus on broadening the scope of subjects, diversifying measurement tools, and emphasizing randomization and generalizability in experimental designs.

**Supplementary Information:**

The online version contains supplementary material available at 10.1186/s12912-024-02172-9.

## Background

The World Health Organization (WHO) defines complementary and alternative medicine (CAM) as healthcare practices outside a country's traditional or conventional medicine [1]. According to the National Center for Complementary and Integrative Health (NCCIH), CAM encompasses nutritional approaches (e.g., herbs), psychological methods (e.g., mindfulness), physical therapies (e.g., massage), integrated mind–body practices (e.g., yoga or auricular acupressure), and modalities that combine psychology and nutrition [2]. This definition suggests CAM may facilitate holistic nursing by addressing both psychological and physical aspects [3]. Consequently, substantial CAM research is conducted in nursing internationally [4, 5], spanning areas like pain, depression, anxiety, chronic disease symptoms, sleep disturbances, and vomiting [4–6]. Classification systems exist, with the Korean Nursing Association (2023) delineating 12 CAM subcategories [6] and NCCIH outlining 76 therapies across major categories like nutrition, body, and psychotherapies [4–7]. The multitude of CAM types has prompted trend identification research, including reviews on Chinese medicine for allergic rhinitis, aromatherapy, auricular acupressure, and CAM for COVID-19 [6, 8]. However, many previous studies have significant limitations in comprehensively identifying overall research trends in CAM. First, they tend to focus narrowly on specific diseases or treatments, lacking a broader perspective on the field as a whole [6, 8]. Second, the use of search queries containing keywords from a specific discipline or arbitrarily selected by researchers introduces bias and hinders the identification of overarching trends [9, 10]. These limitations highlight the need for a more systematic and data-driven approach to analyzing CAM research trends [11–13]. A previous study [14, 15] suggested the use of text mining technique as an approach for literature review [16]. To date, the analysis on research trend in nursing has been conducted more than five years after publication or has only been conducted with partial analyses through literature reviews and text mining [17–19].

The overarching goal was to extract keywords identifying domestic and international CAM research trends using text network analysis and analyze these trends within the nursing field. Specific objectives were: 1) Identify frequency, degree centrality, closeness centrality, and betweenness centrality for keywords appearing in domestic and international CAM studies; 2) Identify key themes within these studies; 3) Discern nursing keywords among sub-topic groups; 4) Analyze and compare nursing and other disciplinary literature based on findings; and 5) Analyze the trend of CAM in nursing based on extracted nursing keywords.

## Methods

### Study design and methodological framework

This study employs a novel methodological framework that combines text mining techniques with expert validation to identify and analyze CAM research trends in a comprehensive and data-driven manner. The framework consists of the following key steps.

1. Data collection: A comprehensive search of multiple databases is conducted to collect a broad range of CAM-related literature across various disciplines.
2. Text preprocessing involves several techniques to prepare the data for analysis. These include natural language processing, stopword removal, and synonym standardization.
3. Keyword extraction and network analysis: Text mining techniques, including term frequency-inverse document frequency (TF-IDF) and centrality analysis, are applied to extract key topics and analyze their relationships within the literature.
4. Topic modeling: Latent Dirichlet Allocation (LDA) is used to identify latent topics within the literature and visualize their proportions and relationships.
5. Expert validation: An expert panel of physicians, nurses, and pharmacists is consulted to validate the relevance and credibility of the identified topics and classify them into respective academic fields.
6. Focused literature analysis: Based on the expert-validated nursing-related topics, a focused re-search and analysis of the literature are conducted to identify trends specific to nursing research on CAM.

**Fig. 1:**
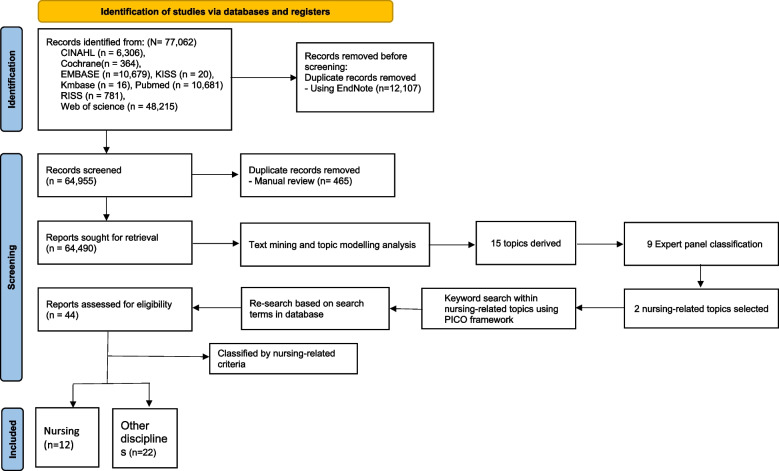
Flow diagram for literature selection process

This multi-step framework allows for a more comprehensive and less biased exploration of CAM research trends by leveraging text mining techniques to process large volumes of literature, identify key topics, and uncover patterns that may not be apparent through traditional review methods [14–16]. The integration of expert validation ensures the relevance and credibility of the findings, while the focused analysis of nursing literature provides insights specific to the nursing discipline within the broader context of CAM research. The process of selecting studies for our analysis is illustrated in Fig. 1, which provides a clear visual representation of the key steps involved, from the initial database search to the final classification of studies into nursing and other disciplines. This multi-step approach, combined with the visual aid, enhances the clarity and transparency of our methodology, allowing readers to better understand and contextualize the subsequent data analysis steps.

### Literature selection

This study focused on complementary and alternative medicine studies conducted in the fields of medicine, public health, and nursing in Korea and abroad. After specifying the research title and abstract as the search scope to extract the literature and build a database, the literature related to nursing was classified based on the topics derived through text network analysis and then, the literature that met the selection criteria was secondarily extracted and analyzed through the abstract screening. The three researchers checked the consistency of the study selection process and if there was any discrepancy, the final decision was made through consensus among the researchers.

1. The selection criteria for the literature were: (1) domestic and foreign studies published within the last five years (January 2018 to September 2023) that conducted studies on complementary and alternative medicine; and
2. The exclusion criteria for the literature were grey literatures, dissertations, and studies for which original texts are not available.

### Data collection strategies

In this study, the database was selected by referring to the COSI (Core, Standard, Ideal) [20] model presented by the National Library of Medicine for literature search. EMBASE, Cochrane Central, and PubMed Central were selected as the core databases.

On the other hand, the standard databases selected were the Cumulative Index to Nursing and Allied Health Literature (CINAHL); and Korean database services such as the Research Information Sharing Service (RISS), KMbase, and Korean studies Information Service System (KISS). These Korean databases were included to ensure a comprehensive coverage of potentially relevant studies published in South Korea, as they index a wide range of domestic and international journals, conference proceedings, and dissertations across various disciplines, including those related to CAM. However, it is important to note that the inclusion of these Korean databases does not limit the scope of our study to Korean literature only, as the majority of our analysis focuses on studies published in English and indexed in the core and standard international databases.

In addition, the Web of Science was selected to include a wider range of literature for the ideal database, and the period of literature search focused on the last five years, from 1 January 2018 to 15 September 2023. to capture the most recent trends in CAM research following the last comprehensive analysis of CAM research trends conducted in 2018 by Sung et al. [19]. This time frame was chosen to provide an updated and comprehensive analysis of CAM research trends, building upon the findings of previous studies and identifying new patterns and areas of focus that have emerged in recent years, given the rapid evolution of CAM research and the increasing integration of CAM into mainstream healthcare.

The data collection procedure was limited for both domestic and foreign studies. In case of foreign studies, ‘English’ was limited as the search language, ‘abstract and title’ were identified as the field, ‘article’ was set as the document form, and the keywords were ‘complementary therap*,’ ‘complementary medicine,’ ‘alternative therap*,’ and ‘alternative medicine.’ For the Korean studies, ‘Korean’ was limited as the search language, ‘abstract and title’ were identified as the field, ‘article’ was set as the document form, and the search keywords used were ‘보완대체,’ ‘대체요법,’ and ‘대체의학.’ In searching for the secondary literature, studies in the field of nursing were presented to a group of nine experts including physicians, nurses, and pharmacists with a master's degree or higher, and then the relevant areas were classified to extract the keywords. These keywords were then used in the text mining search. Topic words, the majority of which were classified as nursing, were re-searched in the collected database. The literature selection and classification process were carried out independently by three researchers and promoted through discussions between the researchers.

### Data analysis process

#### Data extraction

A comprehensive literature search was conducted across eight databases: CINAHL, Cochrane, EMBASE, KISS, Kmbase, PubMed, RISS, and Web of Science. This extensive search yielded a total of 77,062 studies. To ensure the integrity and non-redundancy of our dataset, we employed a rigorous two-step deduplication process. First, we utilized the 'Find Duplicates' function in EndNote software for initial automatic deduplication. This function systematically identifies and groups potential duplicate records based on shared metadata such as title, authors, year, and DOI. Through this automated process, 12,107 duplicate records were identified and removed.

Following the automated process, we conducted a manual review to identify and remove any remaining duplicates that the software might have missed. This careful manual screening allowed us to catch subtle duplicates that automated systems might overlook, such as those with slight variations in titles or author names. Through this manual review, an additional 465 duplicate records were identified and removed. In total, our rigorous two-step deduplication process resulted in the removal of 12,572 duplicate records. Of these, 12,107 were removed through automated deduplication and 465 through manual review. After deduplication, 64,490 unique studies were retained for further analysis. These studies were systematically organized by title and subjected to a thorough text preprocessing phase. During this phase, unstructured words were sorted and cleaned using the social networking program Netminer 4.3.3 and text editor Notepad + + (version 8.5.8).

Also, stopwords such as pronouns, adverbs, and numbers were deleted through natural language processing, while exception list, defined words, and thesaurus were registered. The exception list and thesaurus were selected by the three researchers, and if they failed to reach a unanimous agreement, the keywords were refined through consultation and the abstracts and preambles were reviewed again to examine the context in which the words were used. In case of the exception list, literature search keywords or stopwords such as pronouns, adverbs, numbers, and special symbols were considered, while ‘complementary,’ ‘medicine,’ ‘alternative,’ ‘therapeutic,’ ‘therapy,’ ‘therap,’ ‘therapies,’ ‘the,’ ‘a,’ ‘and,’ ‘of,’ ‘for,’ ‘in,’ ‘to,’ and ‘among’ were excluded. Special symbols like ‘’,:'"()&-?# <  >  + "",‘ were excluded as well. As for defined words, ‘cells → cell,’ ‘effects → effect,’ ‘staphylococcus aureus → staphylococcus,’ ‘aureus → staphylococcus,’ ‘characteristics → characterization,’ ‘efficacy → effect,’ ‘rat → mice,’ ‘radio → radiation,’ ‘systems → system,’ ‘agents → agent,’ ‘activity → activation,’ ‘carcinoma → cancer,’ ‘cases → case,’ ‘mouse → mice,’ ‘practices → practice,’ ‘radio sensitization → radiation,’ ‘years → year,’ ‘α → alpha,’ and ‘β → beta’ were selected, and data sorting for synonyms was performed. As a result of the analysis, a database consisting of 464,625 words was constructed.

#### Data analysis

In this study, text mining and topic modeling analysis were employed using textom and RStudio (4.3) to identify keywords related to CAM. Word analysis, TF-IDF, and degree centrality analysis were performed through text mining, with results presented via visualization. TF-IDF determines if a keyword holds actual significance within a document, as words with high TF and TF-IDF values appear frequently and are more likely keywords or important terms [21, 22]. Following previous studies [22, 23], the minimum word length was set to two, with the top 20 words extracted per topic. Text network analysis created word networks expressing co-occurrence frequency as links [24]. To gauge word occurrence frequency, words were converted to word-word one-mode, and degree centrality analysis identified highly influential network words. The results of these analyses, including frequency, TF-IDF, degree centrality, closeness centrality, and betweenness centrality of core keywords, can be found in Table 1.

**Table 1:** Core keyword by frequency, TF-IDF, degree centrality, closeness centrality, and betweenness centrality

Rank	Keyword									
	Frequency		TF-IDF		Degree centrality		closeness centrality		Betweenness centrality	
	word	value	word	value	word	value	word	value	word	value
1	cell	7653	cell	17,310	effect	1.000	effect	1.000	effect	0.0015
2	patient	6910	effect	17,190	treatment	1.000	treatment	1.000	treatment	0.0015
3	treatment	6851	cancer	16,860	study	1.000	study	1.000	study	0.0015
4	cancer	6722	patient	15,582	analysis	1.000	analysis	1.000	analysis	0.0015
5	study	6295	treatment	15,520	disease	1.000	disease	1.000	disease	0.0015
6	effect	6203	study	14,777	approach	1.000	approach	1.000	approach	0.0015
7	analysis	3618	analysis	10,456	model	0.995	factor	1.000	factor	0.0015
8	disease	3390	disease	10,071	patient	0.990	model	0.995	model	0.0014
9	trial	3244	trial	9789	activation	0.990	patient	0.990	type	0.0014
10	review	2948	review	9115	use	0.990	activation	0.990	activation	0.0014
11	model	2411	activation	9071	evaluation	0.990	use	0.990	patient	0.0014
12	activity	2397	model	7936	drug	0.985	evaluation	0.990	use	0.0014
13	drug	2258	case	7817	cell	0.980	type	0.990	drug	0.0014
14	use	2232	mice	7809	review	0.975	drug	0.985	evaluation	0.0014
15	efficacy	2156	drug	7742	case	0.975	response	0.985	review	0.0014
16	case	2117	use	7558	cancer	0.970	cell	0.980	strategy	0.0014
17	inhibition	1991	inhibition	6979	management	0.965	strategy	0.980	role	0.0014
18	management	1870	management	6632	trial	0.960	review	0.975	development	0.0014
19	health	1731	health	6407	inhibition	0.960	case	0.975	response	0.0013
20	breast	1679	breast	6202	health	0.920	system	0.975	application	0.0013

*TF-IDF* Term Frequency-inverse document frequency

This study utilized Latent Dirichlet Allocation (LDA) for topic modeling, a statistical method that estimates the probability distribution of topics within documents based on the Document Term Matrix (DTM). Following established practices in the literature, we set the Markov Chain Monte Carlo (MCMC) parameters to alpha = 1.44, beta = 0.001, and iterations = 1,000 [25]. To determine the optimal number of topics, we iteratively tested configurations ranging from 1 to 20 topics. Through a combination of silhouette clustering analysis and researcher consensus, we identified that a 15-topic model best represented the research trends in our corpus.

**Fig. 2:**
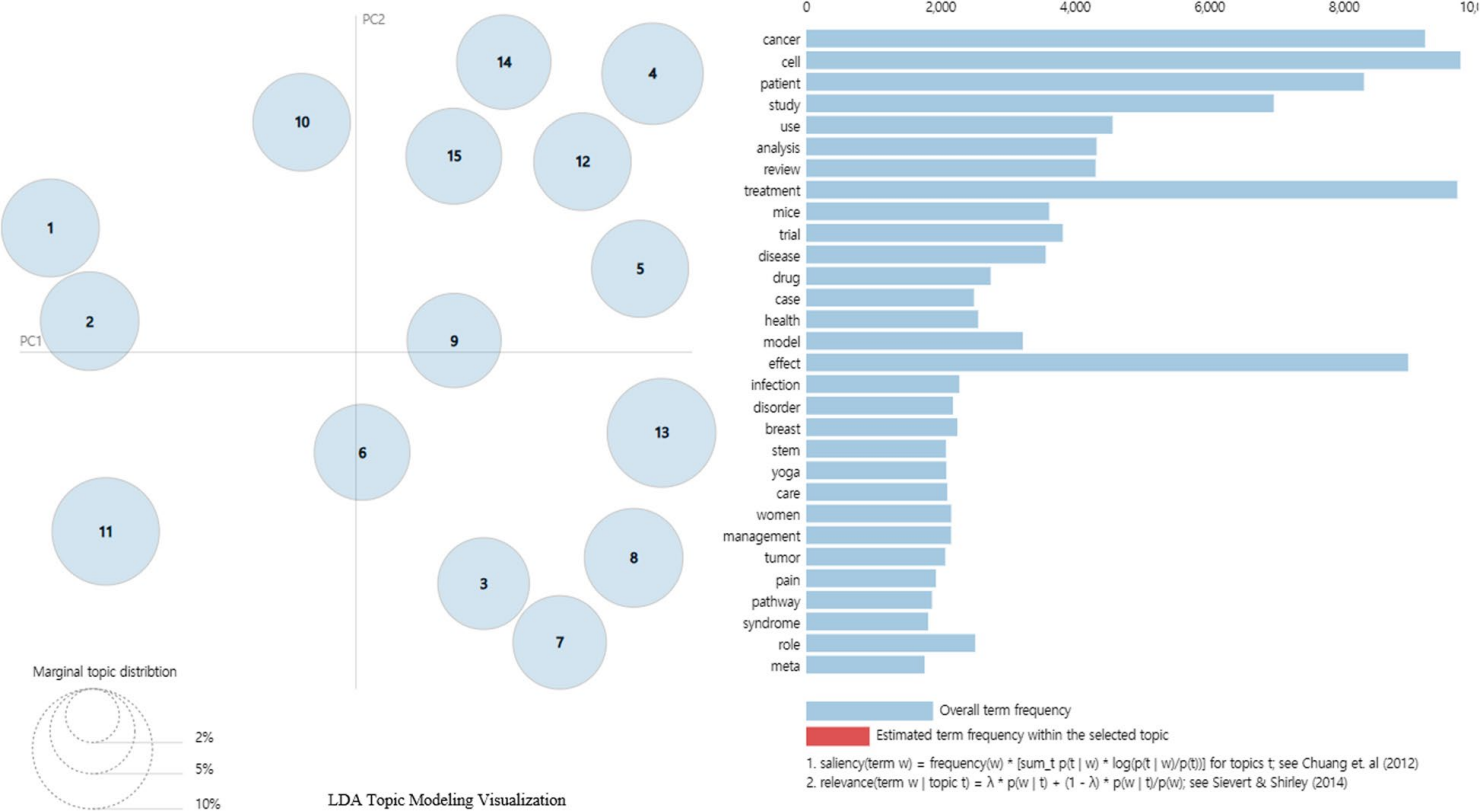
LDA topic modeling visualization

LDA visualization indicated that larger topic sizes represented greater proportions within the analyzed studies [25]. We confirmed that the ideal number of topics, where topics do not overlap and have distinct boundaries, is 15, as shown in Fig. 2. To validate the relevance and credibility of the topic modeling results, we consulted an expert panel consisting of physicians, nurses, and pharmacists with master's or doctoral degrees. The panel members were asked to classify the 15 derived topics into their respective academic fields. Based on the survey results, two topics (Topics 4 and 7) were identified as nursing-related, with the majority of the expert panel categorizing them as such.

Using the words from these two nursing-related topics, a keyword search was conducted within the database to identify the final set of literature containing these terms. The selected literature was then classified as either nursing-related or non-nursing-related based on the following criteria: (1) the study was published by a nursing school or department, (2) the authors were nurses or nursing researchers, (3) the authors were hospital-affiliated nurses, or (4) the study was published in a nursing journal. The classification process was carried out independently by three authors, and the final categorization was determined through a verification process among them.

#### Literature review

After the three researchers re-searched the database built based on the sub-words of the extracted topics, a total of 35 articles were selected, including 13 nursing-related literatures and 22 other discipline-related literatures. The sub-words used for the re-search were derived from Topic 4 and Topic 7 in Table 2 and were classified using the PICO (Population, Intervention, Comparison, Outcome) framework. The population-related sub-words included 'patient,' 'students,' and 'nursing.' The intervention-related sub-words were 'yoga,' 'treatment,' 'radiation,' 'acupuncture,' 'education,' and 'cam.' The comparison-related sub-word was 'placebo,' and the outcome-related sub-words included 'anxiety,' 'depression,' 'symptoms,' 'knowledge,' 'attitudes,' and 'perceptions.' These PICO-classified sub-words were used to conduct the database re-search.

**Table 2:** Latent Dirichlet Allocation(LDA) topic modeling

Key word rank	Topics														
	1	2	3	4	5	6	7	8	9	10	11	12	13	14	15
Expert Topic Classification	Medicine	Medicine	Medicine	Nursing	Medicine	Medicine	Nursing	Pharmacology	Medicine	Pharmacology	Medicine	Medicine	Medicine	Medicine	Medicine
1	model	cell	infection	trial	treatment	type	role	case	syndrome	mice	cancer	analysis	patient	effect	disorder
2	pathway	stem	quality	effect	use	diabetes	survey	health	impact	drug	breast	review	study	wound	extract
3	cell	strategy	risk	yoga	covid	application	practice	women	production	system	tumor	meta	disease	acid	mechanism
4	activation	tissue	life	treatment	children	protein	evidence	care	liver	nanoparticle	inhibition	intervention	outcome	injury	stress
5	beta	line	patient	radiation	combination	approach	failure	management	agent	delivery	factor	function	experience	research	people
6	signaling	method	virus	phage	stimulation	evaluation	utilization	pain	assessment	alpha	lung	development	safety	bone	effect
7	apoptosis	characterization	implications	protocol	gene	profile	heart	report	synthesis	effect	response	protocol	year	healing	spectrum
8	inflammation	cord	alzheimer	anxiety	pregnancy	diagnosis	students	perspective	body	arthritis	cell	transplantation	imaging	hiv	activation
9	lymphoma	decoction	control	dose	prevention	blood	cam	surgery	properties	peptide	chemo	effect	term	skin	post
10	hypertension	transplantation	sleep	zinc	pandemic	plasma	education	treatment	time	metabolism	expression	leukemia	center	brain	communication
11	mise	differentiation	tract	symptoms	vitamin	lipid	healthcare	survival	kidney	learning	growth	network	results	pressure	staphylococcus
12	effect	regeneration	deficiency	depression	nerve	status	valve	series	laser	oil	resistance	stroke	cohort	colitis	fibrosis
13	glioblastoma	serum	oxygen	placebo	design	muscle	knowledge	literature	prevalence	tool	receptor	osteoarthritis	effect	comparison	epilepsy
14	obesity	activation	reality	acupuncture	validation	performance	communication	oncology	compounds	changes	target	decision	data	injection	benefits
15	group	blood	protein	feasibility	approach	challenges	narrative	sars	screening	administration	regulation	knee	bowel	treatment	weight
16	diet	gamma	withdrawal	training	gut	bladder	practitioners	program	characterization	psoriasis	prostate	treatment	exercise	trends	animal
17	induction	matrix	era	insights	adults	surface	attitudes	rna	information	activation	splicing	relationship	hospital	cost	bacteria
18	AKT	vesicles	hepatitis	toxicity	microbiota	vaccine	nursing	massage	species	peptidase	kinase	update	parkinson	dysfunction	self
19	death	adipose	lifestyle	mri	asthma	detection	perceptions	society	immunity	damage	melanoma	music	pilot	exposure	autism
20	proliferation	food	response	emergency	effect	antibiotics	pseudomonas	test	stage	foot	progression	parents	rehabilitation	level	integration

In order to examine the research trends in nursing and other related fields, general characteristics (author, country of publication, year of publication) and research characteristics (research design model, statistical method, research subject, intervention method, outcome variable, measurement instruments) were identified, presented, and compared. Meanwhile, the three researchers independently prepared a characteristic table to ensure the accuracy of the extracted contents and if there was any discrepancy, one data was selected through the discussion process until a consensus was reached and a characteristic table was constructed.

To assess the quality of the selected studies, we employed the Mixed Methods Appraisal Tool (MMAT), a concise tool designed to evaluate various study designs, including qualitative, randomized controlled trials, non-randomized studies, quantitative descriptive studies, and mixed methods studies [26]. This comprehensive tool allowed us to systematically evaluate the methodological rigor of our diverse selection of studies. Each study was evaluated against five MMAT criteria specific to its design, focusing on aspects such as research question appropriateness, data collection methods, and result interpretation. Our assessment revealed varying levels of methodological quality. Among nursing studies (A1-A12), 25% were high quality (5/5 criteria met), 58.3% moderate quality (4/5 criteria), and 16.7% low quality (3/5 criteria). Importantly, all included studies met at least 3 out of the 5 MMAT criteria, indicating an overall moderate to high quality across the selected literature. This suggests that the studies included in our analysis provide a reliable foundation for drawing conclusions. Studies that did not meet all criteria were carefully reviewed, and their potential limitations were considered when interpreting their findings. The MMAT provided a useful overview of study quality and was deemed suitable for assessing methodological rigor while maintaining the feasibility of our analysis. This approach ensured a balanced and nuanced interpretation of the evidence in the field of complementary and alternative medicine. The detailed results of this quality assessment can be found in Tables 3 and 4.

**Table 3:** Description of the studies in nursing

	First author (yr)	Region	Study Design	Sample		Intervention	Statistics	Outcome		MMAT Quality Assessment
				Subject type	Total Size	Category		Dependent	Measurement	
A1	Abdulah (2021)	Turkey	Descriptive	Medical team	794	NA	• Independent t-test • ANOVA • Turkey post hoc test	• Knowledge • Attitude • Use degree	• Holistic complementary and alternative medicine questionnaire	• Moderate quality • (4/5 criteria met)
A2	Ayfer (2021)	Australia	RCT	Nursing student	75	Yoga	• Independent t-test	• Mental symptom • Cortisol	• Brief symptom inventory • Electrochemiluminescence immunoassay	• Moderate quality • (4/5 criteria met)
A3	Emilie (2021)	Germany	Quasi (pre-post)	Medical team	82	Education	• Descriptive • Independent t-test • χ^2^ test • Bonferroni	• Knowledge • Attitude • Preparation • Clinical nursing practice	• The tool developed by the researcher	• Low quality • (3/5 criteria met)
A4	Ezgi (2019)	Germany	RCT	Brest cancer pt	60	Music	• Independent t-test • χ^2^ test • ANCOVA	• Anxiety • Comfortable	• Hospital anxiety and depression scale • Radiation therapy comfortable survey	• High quality • (5/5 criteria met)
A5	Jeanene (2023)	United states of america	Quasi (pre-post)	Cancer Pt	82	Yoga	• Independent t-test	• Fatigue • Pain • Anxiety • Stress	• The tool developed by the researcher	• Moderate quality • (4/5 criteria met)
A6	Jennifer (2023)	United states of america	Quasi (pre-post)	Nurse	23	Education	• Descriptive	• Prevalence • Treatment • Knowledge	• The tool developed by the researcher	• Moderate quality • (4/5 criteria met)
A7	Judith (2020)	United states of america	RCT	HTN Pt	69	Acupressure	• Independent t-test • χ^2^ test • Cohen’s D • RM ANOVA	• Pain • Quality of life	• McGill pain questionnaire • Seattle angina questionnaire-7	• Moderate quality • (4/5 criteria met)
A8	Lei (2022)	United kingdom	RCT	TA PTST Pt (women)	94	Yoga	• Independent t-test • χ^2^ test • Cohen’s D • RM ANOVA	• PTSD symptom • Depression • Anxiety	• Impact of event scale-revised • Depression anxiety stress scales-21	• Moderate quality • (4/5 criteria met)
A9	Lyndall (2021)	United kingdom	Descriptive	Midwife	571	NA	• Descriptive • Independent t-test • χ^2^ test • ANOVA	• Knowledge • Training degree • type • Confidence • Competence	• The tool developed by the researcher	• Moderate quality • (4/5 criteria met)
A10	Özgür (2023)	Turkey	Descriptive	Nurse	122	NA	• Descriptive • Independent t-test • χ^2^ test • ANOVA • Correlation	• Knowledge • Attitude	• The tool developed by the researcher	• Low quality • (3/5 criteria met)
A11	Sandra (2021)	United states of america	Descriptive	Nurse	458	NA	• Descriptive • Independent t-test • χ^2^ test • Fisher’s exac test • ANOVA • Correlation	• Knowledge • Attitude • Belief • Use degree	• The tool developed by the researcher	• Moderate quality • (4/5 criteria met)
A12	Wendy (2020)	United kingdom	Qualitative	Naturopaths	21	NA	• Theme	• Role • Treatment method	• Survey • Interview	• High quality • (5/5 criteria met)

*RCT* Randomized Controlled Trial, *NA* Not Applicable, *χ2 test* Chi-Square Test, *ANOVA* Analysis of Variance, *ANCOVA* Analysis of Covariance, *RM ANOVA* Repeated Measures Analysis of Variance, *HTN* Hypertension

*TA PTST* Trauma and Post-Traumatic Stress Disorder, *pt* Patient, *t-test* t-Distribution Test

**Table 4:** Description of the studies not in the field of nursing

	First author (yr)	Region	Study Design	Sample		Intervention	Statistics	Outcome		MMAT Quality Assessment
				Subject type	Total Size	Category		Dependent	Measurement	
B1	Ahmad (2022)	Hong Kong	RCT	Peripheral artery disease Pt	30	Acupuncture (laser)	• McNemar–Bowker Test • Regression • MANOVA	• Blood flow • Pain	• Edinburgh claudication questionnaire • 6-min walk distance	• Moderate quality • (4/5 criteria met)
B2	Anjana (2020)	India	RCT	Hypertension Pt	80	OM chantinf and yoga	• RM ANOVA • Paired t-test • Independent t-test	• Depression • Anxiety • Stress • Sleep	• Depression anxiety stress scale • Pittsburgh sleep quality index • Heart rate variability	• Moderate quality • (4/5 criteria met)
B3	Chunhui (2022)	China	RCT	Crohn's Pt	66	Acupuncture and moxibustion	• Independent t-test • χ^2^ test • ANOVA • Fisher’s exact test	• Clinical mortality rate • Clinical response rate • Crohn’s disease activity Index and average change in serum c-reactive protein • Steroid-free remission rate • Average change in crohn’s disease endoscopic index of severity score and histopathological score, and the recurrence cumulative rate	• Crohn’s disease activity index • C-reactive protein • Crohn’s disease endoscopic index of severity • Histopathological score • Fecal calprotectin • Operational taxonomic unit • Linear discriminant analysis effect size	• Low quality • (3/5 criteria met)
B4	Conway (2021)	United states of america	Quasi (pre-post)	Hemodialysis Pt	11	Beathing in yoga	• Paired t-test • Wilcoxon • RM ANOVA	• Depression • Anxiety • Quality of life	• Anxiety, depression of survey • Kidney Disease Quality of Life-36 Questionnaire	• High quality • (5/5 criteria met)
B5	Dehghan (2021)	China	RCT	Human immunodeficiency virus Pt	50	Dietary therapy (green tea capsule)	• Generalized linear model • Independent t-test • Fisher’s exact test • Mann–Whitney U test	• Depression	• Hamilton depression scale • Cluster of differentiation 4 cell numbness	• Moderate quality • (4/5 criteria met)
B6	Hai (2019)	United states of america	RCT	After stroke depression Pt	208	Acupuncture	• Descriptive • Independent t-test • χ^2^ test • Wilcoxon • ANOVA	• Depression	• Hamilton depression scale • Self rating depression scale • National institute of health stroke scale • Modified barthel index • Treatment emergent symptom scale • Traditional chinese medicine • stroke diagnosis and evaluation criteria • Clinical global impression • Proton magnetic resonance spectroscopy • Serum	• Moderate quality • (4/5 criteria met)
B7	Hamidreza (2020)	United states of america	RCT	Carpal tunnel syndrome Pt	60	Acupuncture	• Paired t-test • Independent t-test • χ^2^ test • ANOVA	• Pain, numbness, tingling, weakness/clumsiness and night awakenings • Distal motor latency, distal sensory latency, sensory nerve action potential and compound muscle action potential	• Symptom survey • Electrodiagnostic studies	• High quality • (5/5 criteria met)
B8	Huan (2022)	United kingdom	RCT	Pelvic organ prolapse Pt	160	Acupuncture (electro acupuncture)	• Independent t-test • Regression	• Pelvic organ prolapse symptom	• Pelvic floor distress inventory-short form 20 • Pelvic floor impact questionnaire-short form 7 • Pelvic organ prolapse/urinary incontinence sexual questionnaire-short form 12 • International consultation on incontinence questionnaire-urinary incontinence short form • International consultation on incontinence questionnaire-lower urinary tract symptoms quality of life • Patient global impression of improvement • Patient global impression of severity • Patient satisfaction scale	• Moderate quality • (4/5 criteria met)
B9	Jianya (2022)	China	RCT	Insomnia Pt	126	Taichi therapeutic exercise	• Cochran-Mantel–Haenszel • ANOVA • ANCOVA	• Insomnia	• Pittsburgh sleep quality index • Hamilton anxiety scale • Deneralized anxiety disorder 7-item scale • Insomnia seceruty index	• High quality • (5/5 criteria met)
B10	Jo (2018)	United kingdom	RCT	Dementia Pt	60	Aroma massage	• RM ANOVA	• Behavioral and psychological symptoms • Activities of daily living • Cognitive function • Pain	• Cohen-mansfield agitation inventory • Neuropsychiatric inventory • Mini-mental state examination • Barthel Index-20	• High quality • (5/5 criteria met)
B11	Kang (2023)	United states of america	RCT	Parkinson's disease pt	80	Acupuncture	• Independent t-test • ANOVA • Regression • RM ANOVA	• Depression	• Beck depression inventory • Parkinson’s disease sleep scale • State-trait anxiety inventory • The korean version of the fatigue, resistance, ambulation, illnesses, and loss of weight questionnaire scale • Unified parkinson’s disease rating scale III • Functional near-infrared spectroscopy • Health related quality of life • European quality of life visual analogue scale	• High quality • (5/5 criteria met)
B12	Katharina (2023)	United kingdom	RCT	Depression Pt	156	Cognitive behavioral therapy	• Katharina luttenberger	• Depressive symptom severity	• Montgomery-asberg depression rating scale • Patient Health questionnaire	• High quality • (5/5 criteria met)
B13	Lih (2021)	Germany	RCT	Irritable bladder syndrome Pt	66	Meridian point patch	• Independent t-test • Fisher’s exact test • χ^2^ test	• Irritable bladder syndrome	• Overactive bladder symptom score • Bladder awareness • Intensity of urgency scale • Residual urine	• High quality • (5/5 criteria met)
B14	Ling (2022)	United kingdom	RCT	After stroke depression Pt	56	Acupuncture	• Descriptive • χ^2^ test • ANOVA • Mann–Whitney U test • Linear mixed model	• Depression	• Hamilton depression scale-17 • Wisconsin card sorting test • Event-related potentials	• Moderate quality • (4/5 criteria met)
B15	Mehran (2021)	United kingdom	RCT	Migraine Pt	80	Acupuncture	• Independent t-test • χ^2^ test • RMANOVA • ANCOVA	• Frequency of migraine • Duration of mifraine • Pain score • Nausea • Vomiting	• VAS • Satisfaction survey	• Moderate quality • (4/5 criteria met)
B16	Melissa (2022)	Netherlands	RCT	Depression and anxiety Pt	59	Cognitive behavioral therapy	• Linear mixed model	• Depression • Anxiety • Stress	• Depression Anxiety Stress Scale—21	• High quality • (5/5 criteria met)
B17	Naomi (2023)	United states of america	RCT	Anxiety Pt	226	Yoga, Cognitive behavioral therapy and stress education	• Generalized linear mixed model • Mediation effect model	• Response rate	• Clinical global impression–improvement scale • Meta-cognition questionnaire • Five facet mindfulness questionnaire	• High quality • (5/5 criteria met)
B18	Rima (2019)	Netherlands	RCT	Arthritis Pt	72	Yoga	• Independent t-test • ANOVA • wilcoxon • Mann–Whitney U test • Fisher’s exact test • Correlation	• Arthritis	• Disease activity score 28 • Erythrocyte sedimentation rate • Health assessment questionnaire disability index • Beck depression inventory II scale • C-reactive protein • Interleukin-6 • Tumor necrosis factor-alpha	• Moderate quality • (4/5 criteria met)
B19	Seung (2023)	United states of america	Descriptive	PTSD soldier Pt	NA	Acupuncture	• Qualitative Analysis	• PTSD symptom	• PTSD diagnosis index	• High quality • (5/5 criteria met)
B20	Xuan (2022)	United states of america	RCT	Depression and insomnia Pt	270	Acupuncture (electro acupuncture)	• Linear mixed model • ANOVA • Bonferroni	• Sleep • Depression • Anxiety • Insomnia	• Pittsburgh sleep quality index • Hamilton depression scale • Self-rating anxiety scale • Insomnia severity index • Actigraphy-sleep	• High quality • (5/5 criteria met)
B21	Xuan (2023)	United kingdom	RCT	Depression and sleep disorder Pt	270	Acupuncture (electro acupuncture)	• Descriptive • ANOVA • ANCOVA • RM ANOVA	• Depression • Sleep	• Pittsburgh sleep quality index • Actigraphy-sleep • Hamilton rating scale for depression • Self-rating anxiety scale • Treatment emergent symptom scale	• High quality • (5/5 criteria met)
B22	Yuan (2023)	United kingdom	RCT	Gallbladder stone Pt	86	Acupuncture	• ANOVA • RMANOVA • Regression	• Pain intensity • Time points • Gastrointestinal symptoms • Anxiety during pain	• NRS • VAS • Pain anxiety symptoms Scale-20 • Fear of pain questionnaire-III • Pain catastrophizing scale • Acupuncture expectancy scale • Chinese version of the modified massachusetts general hospital acupuncture sensation scale	• High quality • (5/5 criteria met)

*RCT* Randomized Controlled Trial, *Pt* Patient, *MANOVA* Multivariate Analysis of Variance, *RM ANOVA* Repeated Measures Analysis of Variance, *ANOVA* Analysis of Variance, *ANCOVA* Analysis of Covariance, *χ2 test* Chi-Square Test, *VAS* Visual Analogue Scale, *NA* Not Applicable

### Data collection and ethical considerations

Since the data used in this study did not contain information that can identify individuals, the study was conducted after obtaining an IRB approval (IRB No: ewha-202311–0008-01) from the Institutional Review Board of Ewha Womans University.

## Results

### Analysis of word frequency and centrality

The frequency and percentage of the top 20 words related to complementary and alternative medicine are shown in Table 1. The frequency and percentage of the top 20 words related to complementary and alternative medicine are shown in Table 1. The table presents the top 20 keywords ranked by frequency, TF-IDF, degree centrality, closeness centrality, and betweenness centrality. The frequency column indicates the number of times each keyword appears in the analyzed documents, while the TF-IDF column represents the importance of each keyword within the entire document set. Degree centrality, closeness centrality, and betweenness centrality are network analysis measures that indicate the importance and influence of each keyword within the text network. The words with the highest frequency included ‘cell’ (7,653 times), ‘patient’ (6,910 times), ‘treatment’ (6,851 times), ‘cancer’ (6,722 times), ‘study’ (6,295 times), and ‘effect’ (6,203 times). The words with the highest values of TF-IDF, in order, were ‘cell,’ ‘effect,’ ‘cancer,’ ‘patient,’ ‘treatment,’ and ‘study.’ As a result of centrality analysis, the top six common words, in order, were ‘effect,’ ‘treatment,’ ‘study,’ ‘analysis,’ ‘disease,’ and ‘approach.’ Except for common words, the words with the highest values in the centrality analysis, in order, were ‘model,’ ‘patient,’ ‘activation,’ and ‘use.’ The words with the highest values for closeness centrality were ‘factor,’ ‘model,’ ‘patient,’ and ‘activation,’ while the words with the highest values for betweenness centrality were ‘factor,’ ‘model,’ ‘type,’ and ‘activation.’

### Results of the topic modeling

The LDA visualization provides insights into the relative importance and distinctiveness of identified topics. In this visualization, the size of each topic circle is proportional to its prevalence within the analyzed corpus, with larger circles indicating topics that are more frequently discussed across the literature. Interestingly, we observed that some topics, despite being represented by smaller circles, were positioned at considerable distances from other topics. This spatial separation suggests that these topics, while perhaps less prevalent, possess high discriminant validity and represent distinct thematic areas within the field of complementary and alternative medicine research. This interpretation is consistent with established principles in topic modeling, where spatial relationships in visualizations can indicate semantic distinctiveness. An expert panel of 9 individuals (3 doctors, 3 nurses, and 3 pharmacists), each holding a master's or doctoral degree, conducted a survey to classify the topics based on the keywords. The topic that received the most votes from the panel was designated as the representative field for that topic. Based on the resulting values of the topic modeling, 20 sub-words for each topic were presented and provided in Table 2, Topics 1–3, 5–6, and 9–15 were classified as Medicine, Topics 4 and 7 as Nursing, and Topics 8 and 10 as Pharmacology.

The process of selecting studies for our analysis is illustrated in Fig. 2. To determine the optimal number of topics for our analysis, we conducted Latent Dirichlet Allocation (LDA) visualization. As Greene et al. [25] suggest, larger topic sizes in LDA visualization indicate a greater proportion of that topic within the analyzed studies. We tested topic numbers ranging from 1 to 20, seeking a configuration where topics were visually distinct and non-overlapping. This approach aligns with Liu et al. [24], who note that topics with high discriminant validity appear as small but clearly separated clusters. After careful visual analysis, we determined that 15 topics provided the most coherent and distinct groupings, as shown in Fig. 2. This visualization demonstrates the independence and non-overlapping nature of our identified topics, supporting the robustness of our topic modeling approach. Based on the resulting values of the topic modeling, 20 sub-words for each topic were presented and provided in Table 2. The expert panel's classification suggested that Topics 4 and 7 had relevance to nursing research. However, upon closer examination of the keywords included in these topics, it became apparent that they also encompassed literature from other medical disciplines. While the expert panel's classification indicated these topics were nursing-related, the presence of medical terminology suggested a broader interdisciplinary scope. This highlighted the limitations in identifying nursing-specific research using the current topic modeling approach. To address this issue and clarify the nursing-specific research within these topics, a further refinement of the literature search was conducted using the PICO framework. The keywords from Topics 4 and 7 were used to formulate a focused research question and search strategy. This targeted approach yielded a final selection of 12 nursing-specific articles and 22 articles from other disciplines. By employing the PICO framework and leveraging the keywords from the identified nursing-related topics, it was possible to isolate the nursing research within the broader interdisciplinary landscape.

The words included in topic 4 were the following: ‘trial,’ ‘effect,’ ‘yoga,’ ‘treatment,’ ‘radiation,’ ‘phage,’ ‘protocol,’ ‘anxiety,’ ‘dose,’ ‘zinc,’ ‘symptoms,’ ‘depression,’ ‘placebo,’ ‘acupuncture,’ ‘feasibility,’ ‘training,’ ‘insights,’ ‘toxicity,’ ‘mri,’ and ‘emergency.’ The words included in the topic 7 were: ‘role,’ ‘survey,’ ‘practice,’ ‘evidence,’ ‘failure,’ ‘utilization,’ ‘heart,’ ‘students,’ ‘cam,’ education,’ ‘healthcare,’ ‘valve,’ ‘knowledge,’ ‘communication,’ ‘narrative,’ ‘practitioners,’ ‘attitudes,’ ‘nursing,’ ‘perceptions,’ and ‘pseudomonas.’

### Nursing

The characteristics of the 12 studies included in the literature review analysis are shown in Table 3.

Of the 12 final literature selections in nursing, there were four randomized controlled trials [A2] [A4] [A7] [A8], three non-randomized comparative trials [A3] [A5] [A6], four descriptive survey studies [A1] [A9] [A10] [A11], and one qualitative study [A12]. Regarding the country of the study’s publication, there were five studies from the United States, three from the United Kingdom, two from Germany and Turkey, and one from Australia. As for the statistical techniques that appeared with high frequency, 10 studies, which were [A1] [A2] [A3] [A4] [A5] [A7] [A8] [A9] [A10] [A11,] used independent t-test, and it was used in most studies. On the other hand, χ2 test was used in seven studies [A3] [A4] [A7] [A8] [A9] [A10] [A11] and one-way analysis of variance was used in four studies [A1] [A9] [A10] [A11]. Regarding the studies that were conducted targeting patients, there was one study conducted on cancer patients [A5], one study on women with post-traumatic stress disorder caused by a car accident [A8], one study on hypertension patients [A7], and one study on breast cancer patients undergoing chemoradiotherapy [A4]. There were seven studies conducted on medical staffs [A1] [A3] [A6] [A9] [A10] [A11] [A12] and one study conducted on nursing students [A2]. Among the interventional therapies used in clinical trials, the most common one was yoga, which was identified in three studies. Specifically, there was one study that used yoga therapy for chemotherapy patients [A5], laughter yoga for nursing students [A2], and yoga therapy for women with post-traumatic disorder [A8]. There were also studies conducted on virtual cancer education program [A6], education on complementary and alternative medicine [A3], auricular acupressure for hypertensive patients [A7], and music therapy for those with breast cancer [A4]. In the studies conducted among medical professionals and nursing students, knowledge [A1] [A3] [A6] [A9] [A10] [A11], attitudes [A1] [A3] [A10] [A11], and usage surveys [A1] [A11] were identified as measurement variables, whereas depression [A8], pain [A7], quality of life [A7], and anxiety [A8] [A4] were identified as the measurement variables in the studies conducted on patients.

### Other disciplines

The detailed characteristics of these studies, including the study design, sample, intervention, statistical methods, and outcome measures, are presented in Table 4.

Of the 22 final literature selections in other disciplines, there were 20 randomized controlled trials [B1] [B2] [B3] [B5] [B6] [B7] [B8] [B9] [B10] [B11] [B12] [B13] [B14] [B15] [B16] [B17] [B18] [B20] [B21] [B22], one pre- and post-hoc comparative study [B4], and one scoping review [B19]. The detailed characteristics of these studies, including the study design, sample, intervention, statistical methods, and outcome measures, are presented in Table 4. Regarding the country of the study's publication, there were seven studies from the United States of America and the United Kingdom, three studies from China, two studies from the Netherlands, and one study each from Germany, India, and Hong Kong. As for the statistical techniques that appeared with high frequency, there were 10 studies that used independent t-test [B2] [B3] [B5] [B6] [B8] [B11] [B13] [B15] [B18] and one-way ANOVA [B3] [B6] [B7] [B9] [B11] [B14] [B18] [B21] [B20] [B22], while seven studies used repeated measures ANOVA [B2] [B4] [B10] [B11] [B15] [B20] [B22]. All studies for the literature review were conducted on patients. The most common intervention used was auricular acupressure, which was applied on patients with Parkinson’s disease [B11], poststroke depression [B6] [B14], insomnia and depression [B20] [B21], carpal tunnel syndrome [B7], soldiers with PTSD [B19], migraine [B15], pelvic organ prolapse [B8], and gallbladder stones [B22]. The second most common intervention used was yoga therapy, and the subjects were those with active arthritis [B18], generalized anxiety disorder [B17], hemodialysis [B4], and hypertension [B2]. Other subjects and interventions shown in the studies were the following: irritable bladder syndrome patients treated with cinnamon patch [B13]; depression patients treated with bouldering psychotherapy [B12]; dementia patients treated with aromatherapy [B10]; insomnia patients treated with Tai-chi and meridian pressure [B9]; Crohn’s disease patients treated with moxibustion [B3]; HIV patients treated with green tea [B5]; and peripheral arterial disease patients treated with laser acupuncture [B1]. On the other hand, the following were identified as the measurement variables for yoga intervention: level of depression, arthritis stage, anxiety level, quality of life, treatment response rate, sleep, and autonomic function [B2] [B4] [B16] [B17] [B18]. Measurement variables for auricular acupressure included level of depression, sleep quality, level of pain, physical and psychological symptoms, severity of depressive symptoms pelvic organ prolapse, and gastrointestinal symptoms [B3] [B6] [B7] [B8] [B11] [B14] [B15] [B19] [B20] [B21] [B22].

In the study conducted using cinnamon patches, the overactive bladder symptom scores and residual urine volume after urination were identified [B13]. In the study which used green tea, the level of depression was assessed while measuring the severity of depressive symptoms through bouldering [B12]. In the study that used aromatherapy, the behavior, psychology, daily living ability, and cognitive function of the patients with dementia were also assessed [B10].

## Discussion

The present study employed text mining techniques to analyze the literature on CAM published over the past five years and identify trends in nursing research. The text network analysis revealed keywords with high TF-IDF and degree centrality, such as 'cell', 'patient', 'treatment', 'cancer', 'study', and 'effect', suggesting a strong focus on cellular mechanisms, patient-centered approaches, and treatment effects, particularly in the context of cancer [22, 23]. The high centrality of these keywords indicates their importance and influence within the broader network of CAM research [24, 25]. The topic modeling approach identified 15 major topics, providing a comprehensive overview of the key areas of focus in recent CAM research. This data-driven method offers a more nuanced understanding of research trends compared to previous studies that relied on arbitrary searches or focused on narrow populations or interventions [27–31]. By employing this systematic approach, the present study captures the breadth and diversity of CAM research, overcoming the limitations of previous nursing studies.

An expert panel of 9 individuals (3 doctors, 3 nurses, and 3 pharmacists), each holding a master's or doctoral degree, conducted a survey to classify topics based on keywords. According to the expert classification results shown in Table 2, Topics 1–3, 5–6, and 9–15 were classified as Medicine, Topics 4 and 7 as Nursing, and Topics 8 and 10 as Pharmacology. While Topics 4 and 7 were found to be nursing-related, closer examination revealed the presence of literature from other medical disciplines within these topics. To address this issue and clarify the nursing-specific research, a further refinement of the literature search was conducted using the PICO framework. The keywords from Topics 4 and 7 were used to formulate a focused research question and search strategy, yielding a final selection of 34 articles, with 12 nursing-specific articles and 22 articles from other disciplines. Analyzing trends in nursing and interdisciplinary studies within the context of the existing literature provides a more comprehensive understanding of CAM research trends. From a nursing perspective, the identification of topics related to patient care, such as symptom management, quality of life, and patient education, highlights the potential for CAM interventions to improve patient outcomes and experiences. The prominence of keywords such as 'patient', 'treatment', and 'effect' highlights the need for evidence-based practice and the need for rigorous studies to evaluate the efficacy and safety of CAM interventions in nursing care. Furthermore, the expert panel's validation of Topics 4 and 7 as relevant to nursing research emphasizes the relevance of these areas within the nursing discipline. Topic 4, which includes keywords such as 'trial', 'effect', 'yoga', 'anxiety', and 'depression', suggests a focus on the psychological benefits of CAM interventions, particularly in the context of clinical trials. This aligns with the growing recognition of the importance of holistic, patient-centered care in nursing practice [3, 4]. Topic 7, which includes keywords such as 'practice', 'evidence', 'education', 'knowledge', and 'attitudes', highlights the importance of evidence-based practice and the need for nurse education and training in CAM. As CAM interventions become increasingly popular among patients, it is crucial for nurses to have the knowledge and skills needed to provide safe and effective care [5, 6]. The insights gained from this study highlight the potential of text mining and topic modeling techniques for investigating research trends in various fields [11–13]. By leveraging these methods, researchers can systematically analyze large volumes of literature, identify key areas of focus, and uncover patterns and trends that may not be apparent through traditional review methods [14, 15]. This approach can lead to a more comprehensive understanding of the current state of research and inform future directions for investigation.

In conclusion, the present study demonstrates the value of text mining and topic modeling techniques in analyzing research trends, particularly in the field of CAM [9, 10]. The systematic approach employed in this study allowed for a more comprehensive and data-driven exploration of the research landscape, overcoming the limitations of previous studies and providing valuable insights into the trends in nursing research on CAM. The findings of this study have significant implications for nursing practice, highlighting the need for evidence-based approaches, patient-centered care, and the integration of CAM interventions into nursing education and training. Future studies should consider adopting similar methodological approaches to investigate research trends in other fields, as this can lead to a more complete understanding of the current state of research and inform future directions for investigation.

The trends analysis of nursing and interdisciplinary studies on CAM revealed notable differences in research design, subject characteristics, intervention types, and assessment methods. Nursing studies exhibited a more balanced distribution of research designs, including randomized controlled trials [A2, A4, A7, A8], non-randomized comparative trials [A3, A5, A6], descriptive survey studies [A1, A9-A11], and a qualitative study [A12]. In contrast, other disciplines predominantly utilized experimental designs, with 95.2% of the studies being randomized controlled trials [B1-B3, B5-B18, B20-B22]. This disparity suggests that nursing research on CAM should expand its focus on experimental studies to enhance the evidence base and align with the methodological approaches of other disciplines.

The subject characteristics of nursing studies differed significantly from those of other disciplines, with nursing research primarily focusing on healthcare professionals and students [A1, A3, A6, A9-A12], while other disciplines exclusively studied patient populations [B1-B22]. This highlights the need for nursing research to diversify its study subjects and investigate the effects of CAM interventions on patients and healthcare providers [28, 29], as well as broader community and general health populations [3, 6]. By expanding its scope, nursing research can provide valuable insights into the effectiveness and applicability of CAM interventions in promoting health and well-being across diverse settings and populations [4, 5, 7, 8]. Nurses, as frontline healthcare providers, are uniquely positioned to bridge the gap between healthcare settings and the community, engaging with patients and community members to assess their health needs and provide evidence-based recommendations for CAM interventions [1, 2]. This expanded focus, coupled with interdisciplinary collaboration and knowledge exchange [9, 10], can lead to the development of innovative, culturally sensitive, and evidence-based CAM interventions that address the complex health needs of individuals and communities alike.

A closer examination of the intervention types in nursing studies reveals that although they focused on a relatively limited range of CAM modalities, such as yoga [A2, A5, A8] and auricular acupressure [A7], these interventions demonstrated promising potential for managing various symptoms and conditions. For instance, yoga was found to be effective in reducing psychological symptoms and cortisol levels in college students [A2], alleviating chemotherapy-related symptoms in cancer patients [A5], and improving post-traumatic stress disorder among traffic accident survivors [A8]. Similarly, auricular acupressure was shown to help decrease angina symptoms in hypertensive patients [A7]. These research findings suggest that even though the scope of CAM interventions in nursing research may be limited, they can provide significant benefits to diverse patient populations [2, 4, 22]. In contrast, the wide array of CAM interventions investigated in other disciplines, such as aromatherapy for dementia [B10], green tea for depression in HIV patients [B5], laser acupuncture for peripheral arterial disease [B1], cinnamon patch for irritable bladder syndrome [B13], bouldering psychotherapy for depression [B12], Tai-chi and meridian pressure for insomnia [B9], and moxibustion for Crohn's disease [B3], demonstrates the potential for nursing research to explore and apply new therapies. The safety, efficacy, and potential of these diverse CAM modalities, as evidenced in other disciplines [23, 24], should encourage nursing researchers to investigate their applicability in patient care. By conducting rigorous studies on the safety and efficacy of various CAM interventions, nursing research can provide valuable evidence to support the integration of complementary therapies into nursing practice [2, 4, 22]. Moreover, this trends analysis emphasizes the importance of studying CAM interventions for chronic disease management. With the increasing prevalence of chronic conditions [1, 9, 10], nursing research can play a pivotal role in evaluating the effectiveness of CAM for managing these diseases. Studies on yoga for hypertension [B2] and arthritis [B18], auricular acupressure for insomnia and depression [B20, B21], and moxibustion for Crohn's disease [B3] demonstrate the potential of CAM in improving patient outcomes and quality of life. As nurses have more direct and prolonged contact with patients compared to other healthcare professionals, they are well-positioned to assess the effectiveness of CAM interventions in both clinical and community settings [3, 5]. By conducting well-designed studies on the safety and efficacy of various CAM modalities, nursing research can provide the necessary evidence to support the integration of complementary therapies into chronic disease management plans, ultimately enhancing patient care and outcomes across diverse settings. Leveraging their unique role in patient care and conducting rigorous studies on the safety and efficacy of various CAM interventions, particularly for chronic disease management, can enable nursing research to make significant contributions to the integration of complementary therapies into nursing practice. This approach has the potential to not only improve patient outcomes and experiences but also strengthen the evidence base for CAM in healthcare, fostering interdisciplinary collaboration in CAM research and advancing the field of nursing.

The analysis of assessment methods revealed that nursing studies heavily relied on self-developed measurement instruments (58.3%) [A3, A5, A6, A9-A12], while other disciplines predominantly used previously validated tools [B1-B22]. Furthermore, nursing studies rarely incorporated physiological indicators (8.3%) [A2], in contrast to the more frequent use of such measures in other disciplines (36.3%) [B1-B22]. These findings underscore the importance of utilizing validated assessment tools and physiological indicators in nursing research to enhance the reliability and validity of study results [31]. By incorporating these objective measures, nursing research can more clearly identify significant factors and strengthen the level of evidence, ultimately improving the credibility and applicability of the results.

The trends analysis of statistical techniques revealed a higher prevalence of independent t-tests in nursing research (83.3%) [A1-A5, A7-A11], while other disciplines showed a more balanced use of various techniques, including one-way ANOVA (45.5%) [B3, B6, B7, B9, B11, B14, B18, B20-B22] and repeated measures ANOVA (31.8%) [B2, B4, B10, B11, B15, B20, B22]. This difference can be attributed to the nature of the dependent variables assessed in each field, with nursing studies primarily focusing on single assessments of knowledge, attitudes, education, beliefs, and symptoms [A1, A3-A11], whereas other disciplines frequently employed repeated measures of pain, depression, response rate, serum levels, and neurological outcomes [B2-B4, B6-B8, B10, B11, B14-B22]. These findings underscore the importance of aligning the choice of statistical techniques with the nature of the outcome measures to ensure the validity and reliability of the research findings.

In conclusion, the trends analysis of nursing and interdisciplinary studies on CAM highlights the need for nursing research to expand its focus on experimental designs, diversify study subjects, explore various CAM interventions, utilize validated assessment tools and physiological indicators, and employ robust statistical techniques. By addressing these methodological considerations, nursing research can strengthen the evidence base for CAM interventions, facilitate their integration into nursing practice, and contribute to interdisciplinary dialogue in the field of CAM research [11–13]. As CAM use becomes increasingly prevalent among patients, particularly those with chronic conditions [1, 9, 10], nursing research has a crucial role to play in investigating the safety and efficacy of various CAM modalities [2, 4, 22]. This approach not only has the potential to improve patient outcomes and experiences but also enables nursing research to make valuable contributions to interdisciplinary collaboration in the field of CAM [3, 5]. By embracing the diversity of CAM interventions and fostering interdisciplinary interactions, nursing research can broaden its scope, enhance the efficiency of patient-focused care, and move closer to providing truly holistic care that addresses the multifaceted needs of patients. Also, the integration of CAM into nursing practice, supported by robust research evidence, has the power to transform healthcare delivery and improve the lives of patients, particularly those with chronic conditions who stand to benefit greatly from a more comprehensive and individualized approach to care.

The trends analysis of nursing and interdisciplinary studies on CAM highlights the potential for nursing research to draw inspiration from the diverse CAM interventions studied in other disciplines and adapt them for nursing practice. For example, the use of aromatherapy for dementia [B10], green tea for depression in HIV patients [B5], and cinnamon patch for irritable bladder syndrome [B13] could be explored in nursing research to assess their feasibility and effectiveness in nursing care settings. By learning from the experiences of other disciplines and adapting promising CAM interventions for nursing practice, researchers can expand the scope of nursing research on CAM and contribute to the development of innovative, evidence-based complementary therapies for various patient populations. Given the current trends in nursing research on CAM, it is essential for future studies to consider the research directions and methodologies employed in other disciplines to guide the advancement of nursing science in this field. In summary, this trends analysis emphasizes the need for nursing research to embrace a more diverse and rigorous approach to CAM research, drawing inspiration from the methodologies and interventions studied in other disciplines. By expanding the focus on experimental designs, diversifying study subjects, exploring novel CAM interventions, utilizing validated assessment tools and physiological indicators, nursing research can strengthen the evidence base for CAM interventions, facilitate their integration into nursing practice.

## Limitations

This study aimed to identify research trends in CAM through text network analysis and to analyze nursing research trends based on the findings. The use of text mining and big data analysis allowed for a more comprehensive and less biased approach to data collection and processing compared to arbitrary search strategies. However, there were still limitations in defining each field intuitively due to the diverse and wide-ranging areas of CAM used in different disciplines. Future studies should focus on analyzing overall topics across various fields as well as keyword extraction through text mining to gain a more holistic understanding of CAM research trends. Another limitation of this study is that the search languages were restricted to Korean and English. This may have excluded relevant studies published in other languages and might limit the generalizability of the findings. As CAM is rooted in diverse cultures and traditions worldwide, it is important to include studies conducted in various languages for a comprehensive understanding. Future research should incorporate more languages to provide a global perspective on CAM research trends.

Despite these limitations, this study offers a novel methodological strategy for trend analysis by combining keywords extracted using big data rather than relying on researchers' arbitrary settings. The keyword-based classification and literature analysis provide a new approach to identifying research trends and directions. The trends analysis between nursing literature and other disciplines revealed differences in subject selection, study design, statistical techniques, and measurement of dependent variables, highlighting the need for nursing research to broaden the range of subjects and measurement tools while considering randomization and generalization in experimental designs. Furthermore, this study emphasizes the importance of using design techniques that facilitate the sharing of research results beyond the nursing community.

## Conclusions

This study significantly advances CAM research in nursing by providing a comprehensive, data-driven overview of research trends. We have identified key areas for improvement, such as the need for more randomized controlled trials and broader subject diversity, and have proposed innovative methodological strategies. Our findings underscore the importance of interdisciplinary collaboration and the adoption of diverse, rigorous research approaches. By addressing these gaps, nursing research in CAM can be strengthened, ultimately enhancing the integration of evidence-based CAM practices in nursing care and improving patient outcomes.

### Supplementary Information

Supplementary Material 1.

Supplementary Material 2.

## Data Availability

The data and materials of this study are available from the corresponding author upon reasonable request.

## Abbreviations

CAM: Complementary and Alternative Medicine
WHO: World Health Organization
NCCIH: National Center for Complementary and Integrative Health
RISS: Research Information Sharing Service
KISS: Korean studies Information Service System
CINAHL: Cumulative Index to Nursing and Allied Health Literature
TF-IDF: Term Frequency-Inverse Document Frequency
LDA: Latent Dirichlet Allocation
MCMC: Markov Chain Monte Carlo
DTM: Document Term Matrix
MMAT: Mixed Methods Appraisal Tool
IRB: Institutional Review Board
