# Supplementary material for: Literature review of complementary and alternative therapies: using text mining and analysis of trends in nursing research
Source: BMC Nurs. 2024 Aug 1;23:526. doi: 10.1186/s12912-024-02172-9 (PMC11292993; doi:10.1186/s12912-024-02172-9)
Supplement: Supplementary file 1 — Supplementary Material 1. [file 12912_2024_2172_MOESM1_ESM.docx]

Appendix 1. Search Strategy Used in Each Database

EMbase

| Searches | | Result |
| --- | --- | --- |
| #1 | ('complementary therap*':ab,ti OR 'complementary medicine':ab,ti OR 'alternative therap*':ab,ti OR 'alternative medicine':ab,ti) AND [2018-2023]/py | 16,876 |
| #2 | Duplication | 5667 |
| #3 | Manual Duplication | 34 |
| #4 | Retractions | 496 |
| #5 | #1 AND #2 AND #3 | 10,679 |

Cochrane

| Searches | | Result |
| --- | --- | --- |
| #1 | ((((complementary therap*) OR (complementary medicine) OR (alternative therap*) OR (alternative madicine)))) | 364 |

PubMed Central

| Searches | | Result |
| --- | --- | --- |
| #1 | (complementary therap*) OR (complementary medicine) OR (alternative therap*) OR (alternative madicine) "2018/01/01"[Date - Publication] | 10,867 |
| #2 | English | 10,681 |

CINAHL

| Searches | | Result |
| --- | --- | --- |
| #1 | (complementary therap*) OR (complementary medicine) OR (alternative therap*) OR (alternative madicine) “DT 2018-2023” | 6,463 |
| #2 | English | 6,306 |

Web of Science

| Searches | | Result |
| --- | --- | --- |
| #1 | (TI=(complementary therap*)) OR (AB=(complementary therap*)) OR (TI=(complementary medicine)) OR (AB=(complementary medicine)) OR (TI=(alternative therap*)) OR (AB=(alternative therap*)) OR (TI=(alternative medicine)) OR (AB=(alternative medicine)) OR (TI=(complementary medicine)) OR (AB=(complementary medicine)) | 48,215 |

RISS

| Searches | | Result |
| --- | --- | --- |
| #1 | Title (보완대체 OR 대체요법 OR 대체의학) | 109 |
| #2 | Abstract (보완대체 OR 대체요법 OR 대체의학) | 1213 |
| #3 | Duplication | 541 |
| #4 | #1 AND #2 AND #3 | 781 |

KMbase

| Searches | | Result |
| --- | --- | --- |
| #1 | Title (보완대체 OR 대체요법 OR 대체의학) | 9 |
| #2 | Abstract (보완대체 OR 대체요법 OR 대체의학) | 15 |
| #3 | Duplication | 8 |
| #4 | #1 AND #2 AND #3 | 16 |

KISS

| Searches | | Result |
| --- | --- | --- |
| #1 | Title (보완대체 OR 대체요법 OR 대체의학) | 10 |
| #2 | Abstract (보완대체 OR 대체요법 OR 대체의학) | 16 |
| #3 | Duplication | 6 |
| #4 | #1 AND #2 AND #3 | 20 |

Appendix 2. Selected Literature

**(1) The Studies in Nursing**

[A1] Sarman A, Uzuntarla Y, 2022. Attitudes of healthcare workers towards complementary and alternative medicine practices: A cross-sectional study in Turkey. European Journal of Integrative Medicine, 49, 102096.

[A2] Ozturk FO, Tezel A, 2021. Effect of laughter yoga on mental symptoms and salivary cortisol levels in first‐year nursing students: A randomized controlled trial. International Journal of Nursing Practice, 27(2), e12924.

[A3] Hayward EN, Watling CZ, Balneaves LG, 2021. A pre-post evaluation of oncology healthcare providers’ knowledge, attitudes, and practices following the implementation of a complementary medicine practice guideline. Supportive Care in Cancer, 29(12), 7487-7495.

[A4] Karadag E, Uğur Ö, Çetinayak O, 2019. The effect of music listening intervention applied during radiation therapy on the anxiety and comfort level in women with early-stage breast cancer: A randomized controlled trial. European Journal of Integrative Medicine, 27, 39-44.

[A5] Robison J, Walter T, Godsey JA, Robinson J, 2023. Chairside Yoga Therapy Alleviates Symptoms in Patients Concurrently Receiving Outpatient Cancer Infusions: A Promising Feasibility Study. Journal of Holistic Nursing.

[A6] Hotchkiss J, Bula AK, Zimba C, Bingo S, Chinkhata M, Song L, Bryant AL, 2023. Evaluating the Knowledge Change Before and After Continuing Cancer Education in Malawian Nurses. Journal of Cancer Education.

[A7] Schlaeger J, Cai HY, Steffen AD, Angulo V, Shroff AR, Briller JE, DeVon HA, 2019. Acupuncture to improve symptoms for stable angina: protocol for a randomized controlled trial. JMIR Research Protocols, 8(7), e14705.

[A8] Yi L, Lian Y, Ma N, Duan N, 2022. A randomized controlled trial of the influence of yoga for women with symptoms of post-traumatic stress disorder. Journal of Translational Medicine, 20(1), 162.

[A9] Mollart L, Stulz V, Foureur M, 2021. Midwives knowledge and education/training in complementary and alternative medicine (CAM): A national survey. Complementary Therapies in Clinical Practice, 45, 101473.

[A10] Özgür TUG, ŞAHİN SY, İyigün E, 2023. Investigation of knowledge and attitudes of nurses continuing postgraduate education regarding traditional and complementary medicine practices. Sağlık Akademisyenleri Dergisi, 10(3), 433-444.

[A11] Siedlecki SL, 2021. Complementary and alternative therapies (CAT) in academic programs and nursing practice. International Journal of Environmental Research and Public Health, 18(3), 1017.

[A12] Ericksen-Pereira W, Roman NV, Swart R, 2020. The effect of legislation on the treatment practices and role of naturopaths in South Africa. BMC Complementary Medicine and Therapies, 20(1), 1-8.

**(2) The Studies not in the Field of Nursing**

[B1] Ahmad AM, Abdel-Aziz HA, 2022. Laser acupuncture for claudication symptoms in peripheral artery disease—Does it work? A randomized trial. Hong Kong Physiotherapy Journal, 42(01), 31-40.

[B2] Rajagopalan A, Krishna A, Mukkadan JK, 2022. Effect of Om chanting and Yoga Nidra on depression anxiety stress, sleep quality and autonomic functions of hypertensive subjects–a randomized controlled trial. Journal of Basic and Clinical Physiology and Pharmacology, 34(1), 69-75.

[B3] Bao C, Wu L, Wang D, et al., 2022. Acupuncture improves the symptoms, intestinal microbiota, and inflammation of patients with mild to moderate Crohn's disease: A randomized controlled trial. EClinicalMedicine, 45.

[B4] Conway F, Desta MN, Jung YS, Levine DM, Bohmart A, 2021. Intradialytic yoga-based breathing and relaxation to improve anxiety, depression, and quality of life: A pilot feasibility study. Journal of the American Society of Nephrology, 292-292.

[B5] Ali DMS, Alireza MS, Reza SM, et al., 2021. Effect of green tea consumption in treatment of mild to moderate depression in Iranian patients living with HIV: A double-blind randomized clinical trial. Chinese Herbal Medicines, 13(1), 136-141.

[B6] Lu H, Li M, Zhang B, et al., 2019. Efficacy and mechanism of acupuncture for ischemic poststroke depression: study protocol for a multicenter single-blinded randomized sham-controlled trial. Medicine, 98(7).

[B7] Hasanabadi H, Jokar MH, Iranmanesh A, 2020. Acupuncture for carpal tunnel syndrome: a randomized controlled trial studying changes in clinical symptoms and electrodiagnostic tests. Alternative Therapies in Health and Medicine, 26(2), 10-16.

[B8] Chen H, Liu X, Yan Y, et al., 2022. Effect of electroacupuncture on symptoms of female pelvic organ prolapse (stage II–III)(EAPOP study): protocol of a randomised controlled trial. BMJ Open, 12(6), e051249.

[B9] Deng J, Liu X, Wang Y, et al., 2022. The therapeutic effect of Taijiquan combined with acupoint pressing on the treatment of anxiety insomnia in college students: A study protocol for a randomized controlled trial. Frontiers in Psychiatry, 13, 961513.

[B10] Fung JKKM, Tsang HWH, 2018. Management of behavioural and psychological symptoms of dementia by an aroma‐massage with acupressure treatment protocol: A randomised clinical trial. Journal of Clinical Nursing, 27(9-10), 1812-1825.

[B11] Kang DH, Kim JY, Park YC, Yoo HR, Jung IC, 2023. Efficacy and safety of a combination of emotional freedom technique with acupuncture versus acupuncture alone to treat psychiatric symptoms in Parkinson’s disease: A protocol for a randomized, assessor-blind, parallel-group clinical trial. Medicine, 102(21).

[B12] Luttenberger K, Karg‐Hefner N, Berking M, et al., 2022. Bouldering psychotherapy is not inferior to cognitive behavioural therapy in the group treatment of depression: A randomized controlled trial. British Journal of Clinical Psychology, 61(2), 465-493.

[B13] Chen LL, Shen YC, Ke CC, et al., 2021. Efficacy of cinnamon patch treatment for alleviating symptoms of overactive bladder: a double-blind, randomized, placebo-controlled trial. Phytomedicine, 80, 153380.

[B14] Chen L, Chen Y, Wu L, et al., 2022. Efficacy of acupuncture on cognitive function in poststroke depression: study protocol for a randomized, placebo-controlled trial. Trials, 23(1), 85.

[B15] Habibabadi MR, Ashtari F, Raeisi I, 2021. Effect of auricular acupuncture with semi-permanent ear needles on controlling migraine symptoms: a single-blind randomized clinical trial.

[B16] O'Shea M, Capon H, Skvarc D, et al., 2022. A pragmatic preference trial of therapeutic yoga as an adjunct to group cognitive behaviour therapy versus group CBT alone for depression and anxiety. Journal of Affective Disorders, 307, 1-10.

[B17] Simon NM, Hofmann SG, Rosenfield D, et al., 2021. Efficacy of yoga vs cognitive behavioral therapy vs stress education for the treatment of generalized anxiety disorder: a randomized clinical trial. JAMA Psychiatry, 78(1), 13-20.

[B18] Gautam S, Tolahunase M, Kumar U, Dada R, 2019. Impact of yoga based mind-body intervention on systemic inflammatory markers and co-morbid depression in active Rheumatoid arthritis patients: A randomized controlled trial. Restorative Neurology and Neuroscience, 37(1), 41-59.

[B19] Seung HB, Leem J, Kwak HY, et al., 2023. Acupuncture for military veterans with posttraumatic stress disorder and related symptoms after combat exposure: Protocol for a scoping review of clinical studies. PLoS One, 18(4), e0273131.

[B20] Yin X, Dong B, Liang T, et al., 2019. Efficacy and safety of electroacupuncture on treating depression-related insomnia: a study protocol for a multicentre randomised controlled trial. BMJ Open, 9(4), e021484.

[B21] Yin X, Li W, Liang T, et al., 2022. Effect of electroacupuncture on insomnia in patients with depression: a randomized clinical trial. JAMA Network Open, 5(7), e2220563.

[B22] Zhou Y, Shen Y, Ye X, et al., 2023. Acupuncture on GB34 for immediate analgesia and regulating pain-related anxiety for patients with biliary colic: a protocol of randomized controlled trial. BMC Complementary Medicine and Therapies, 23(1), 224.
